# Videokonsultationen durch Psychotherapeuten in Zeiten der COVID-19-Pandemie: Wirksamkeit, Gestaltung des Settings und erste Erfahrungen aus einer Machbarkeitsstudie sowie mit dem Routineangebot im Krankenhaus

**DOI:** 10.1007/s00278-020-00438-6

**Published:** 2020-05-26

**Authors:** Markus W. Haun, Mariell Hoffmann, Justus Tönnies, Ulrike Dinger, Mechthild Hartmann, Hans-Christoph Friederich

**Affiliations:** Klinik für Allgemeine Innere Medizin und Psychosomatik, Zentrum für Psychosoziale Medizin, Universitätsklinikum Heidelberg, Im Neuenheimer Feld 410, 69120 Heidelberg, Deutschland

**Keywords:** Gesundheitsversorgung, Telemedizin, Behandlungswirksamkeit, Behandlungs-Setting, Versorgungsforschung, Delivery of health care, Telemedicine, Treatment efficacy, Treatment setting, Health services research

## Abstract

Wegen der durch die „corona virus disease 2019“ (COVID-19) ausgelösten Pandemie und den resultierenden Beeinträchtigungen persönlicher (d. h. von Angesicht zu Angesicht stattfindender) Behandlung haben Videokonsultationen in der Erbringung von Gesundheitsleistungen massiv an Bedeutung zugenommen. Die meisten Psychotherapeuten haben allerdings bis dato wenig praktische Erfahrung in der Durchführung von Videokonsultationen, nicht zuletzt auch aufgrund bisher eingeschränkter Möglichkeiten zur Abrechnung mit den Kostenträgern. Der vorliegende Beitrag stellt (1) eine Übersicht über die Wirksamkeit per Videokonsultation durchgeführter psychotherapeutischer Interventionen bei depressiven und Angststörungen, (2) Empfehlungen zur spezifischen Gestaltung des Behandlungsrahmens sowie (3) erste Erfahrungen von Patienten und Psychotherapeuten aus einer deutschen Machbarkeitsstudie sowie mit dem Routineangebot im Krankenhaus während der COVID-19-Pandemie vor.

## Abstract (en)

Due to the pandemic caused by the coronavirus disease 2019 (COVID-19) and the resulting constraints on personal (i.e. face to face) treatment, video consultations have recently gained a major role in the delivery of healthcare services; however, until now, most psychotherapists have little experience with conducting video consultations, not least because of poor possibilities for reimbursement from the statutory health insurance. This article provides (1) an overview of the effectiveness of psychotherapy interventions delivered via video consultations for depression and anxiety disorders, (2) recommendations for setting up and conducting these consultations and (3) first experiences of psychotherapists from a German feasibility study and from the provision in routine care in hospital during the COVID-19 pandemic.

Um in Zeiten der durch die „corona virus disease 2019“ (COVID-19) ausgelösten Pandemie einerseits dem (z. T. gestiegenen) Bedarf nach psychotherapeutischer Versorgung nachzukommen sowie andererseits Patienten und Therapeuten keinen zusätzlichen gesundheitlichen Risiken auszusetzen, bieten sich Videokonsultationen in der Psychotherapie an. Diese können hinsichtlich Akzeptanz durch die Patienten und Wirksamkeit vergleichbare Erfolge erzielen wie eine Psychotherapie im klassischen persönlichen Setting. Eine der Voraussetzungen hierfür stellt ein professionelles Behandlungs-Setting dar.

Angesichts der durch die COVID-19-Pandemie resultierenden Beeinträchtigungen persönlicher (d. h. von Angesicht-zu-Angesicht stattfindender) Behandlung kommen Videokonsultationen als Gesundheitsleistungen mehr und mehr zum Einsatz (Greenhalgh et al. 2020; Mann et al. 2020; Mohr et al. 2018; Tuerk et al. 2018). Videokonsultationen bieten sich neben Telefonaten insbesondere in der ambulanten psychotherapeutischen Versorgung an, da

- die technologische Entwicklung mittlerweile eine hohe Bildqualität bei vergleichsweise stabiler Konnektivität ermöglicht,
- Datensicherheitsstandards implementiert wurden und
- für den Bereich psychischer Störungen bereits Evidenz zu Machbarkeit, Wirksamkeit und Patientensicherheit vorliegt (Wright und Caudill 2020).

Unabhängig davon dürften die meisten Psychotherapeuten bis dato wenig praktische Erfahrung mit Videokonsultationen haben, nicht zuletzt auch aufgrund bisher eingeschränkter Möglichkeiten zur Abrechnung mit den Kostenträgern (Kannarkat et al. 2020; Wind et al. 2020). Der vorliegende Beitrag stellt (1) eine Übersicht über die Wirksamkeit videobasierter psychotherapeutischer Interventionen, (2) Empfehlungen zur spezifischen Gestaltung des Behandlungsrahmens sowie (3) erste Erfahrungen von Patienten und Psychotherapeuten aus einer deutschen Machbarkeitsstudie und mit dem Routineangebot im Krankenhaus während der COVID-19-Pandemie vor.

## Wirksamkeit psychotherapeutischer Videokonsultationen bei Depressionen und Angststörungen

Die Wirksamkeit von psychotherapeutischen Interventionen, die per Videokonsultation durchgeführt werden, wurde für depressive und Angststörungen zuletzt systematisch von Yellowlees et al. (2018) untersucht. Bezüglich der Behandlung von Depressionen per Videokonsultation wurden in einer Übersichtsarbeit 14 randomisierte kontrollierte Studien (RCT) identifiziert, in denen jeweils die Kontrollgruppe (Angesicht-zu-Angesicht) die gleiche Intervention (meist kognitive Verhaltenstherapie) erhielt wie die Videokonsultationsgruppe (Berryhill et al. 2019a). In den meisten Studien ergaben sich keine statistisch signifikanten Unterschiede zwischen Videokonsultations- und Kontrollgruppen. In einer Studie fand sich in der Postmessung eine stärkere Reduktion der Symptomlast für die Kontrollgruppe, die jedoch in der Katamnese nicht mehr beobachtet wurde. Einschränkend sollte festgehalten werden, dass die meisten Studien bei tendenziell eher kleinen Stichprobenzahlen vergleichsweise kurze Beobachtungszeiträume wählten und die Hälfte der Studien im Kontext der psychosozialen Versorgung von US-Soldaten durchgeführt wurde. Die Autoren diskutieren einen möglichen Publikationsbias nicht. In einer weiteren Übersichtsarbeit wurden 4 RCT mit Patienten mit posttraumatischen Belastungsstörungen (PTBS) und ein RCT mit einer kleinen Stichprobe von Angststörungspatienten identifiziert (Berryhill et al. 2019b). Die meisten Interventionen beinhalteten kognitive Verhaltenstherapie, Verhaltensaktivierung oder Expositionstherapie. Die Autoren sahen für beide Störungsbereiche keine signifikanten Unterschiede in der Wirksamkeit zwischen per Videokonsultationen und konventionell durchgeführten Psychotherapien.

Ein viel diskutierter Aspekt von psychotherapeutischen Interventionen per Videokonsultationen betrifft das therapeutische Arbeitsbündnis zwischen Psychotherapeut und Patient als wesentlichem Wirkfaktor (Berger 2017; Norcross und Wampold 2018). Häufig wird eingewandt, dass das therapeutische Arbeitsbündnis in Videokonsultationen etwa aufgrund des limitierten Einbezugs nonverbaler Äußerungen vergleichsweise schlechter etabliert werden könne und somit Therapieerfolge weniger wahrscheinlich würden (Hoffmann et al. in Vorbereitung; Stamm 1998). In einer Nichtunterlegenheitsmetaanalyse, die 12 Studien einbezog, wurde tatsächlich eine leichte Unterlegenheit der Videokonsultationsmodalität gegenüber dem klassischen Angesicht-zu-Angesicht-Setting festgestellt, die jedoch nicht signifikant ausfiel (Norwood et al. 2018). In einzelnen Studien fanden sich Divergenzen bezüglich des Arbeitsbündnisses zwischen Patienten und Psychotherapeuten dahingehend, dass Patienten dieses als besser einschätzten. Hinsichtlich der Symptomreduktion erwiesen sich Videokonsultationen in dieser Übersicht als ebenbürtig gegenüber dem Angesicht-zu-Angesicht-Setting. In einer zweiten Übersichtsarbeit mit 23 quantitativen und qualitativen Studien zum therapeutischen Arbeitsbündnis bei videobasierter Psychotherapie kamen Simpson und Reid (2014) zu der Überzeugung, dass Patienten – unabhängig von ihrer Diagnose – das Gefühl von Bindung und Präsenz als mindestens so hoch beschrieben wie in konventionellen Settings. Psychotherapeuten bewerteten das Arbeitsbündnis in der frühen Behandlungsphase demgegenüber niedriger.

Zusammenfassend lässt sich festhalten, dass psychotherapeutische Interventionen per Videokonsultationen hinsichtlich Symptomminderung zu vergleichbaren Ergebnissen führen wie Psychotherapie im klassischen persönlichen Setting. Unabhängig davon liegen derzeit keinerlei Anhaltspunkte dafür vor, dass Videokonsultationen unerwünschte Ereignisse bei Patienten auslösen.

## Gestaltung des Settings

Orientierung zur Durchführung von psychotherapeutischen Videokonsultationen bieten die Empfehlungen der American Psychiatric Association (APA), der American Telemedicine Association (ATA; Shore et al. 2018) sowie lokal der Bundespsychotherapeutenkammer (BPtK; https://www.bptk.de/neue-praxis-info-videobehandlung/). Laut den publizierten Studien unterscheiden sich die Kontraindikationen für Videokonsultationen nicht von jenen im konventionellen Setting. Bei psychotischen Symptomen (insbesondere Fremdbeeinflussungsideen/-wahn) sowie (älteren) Patienten mit Visus- und/oder Hörminderung und/oder kognitiven Defiziten sollte der Einsatz kritisch geprüft werden. Wichtigste Voraussetzung für eine Behandlung ist die Bereitschaft des Patienten, sich auf Videokonsultationen einzulassen. Es empfiehlt sich, beim Erstkontakt mit dem Patienten einen Notfallplan für a) einen alternativen Kontaktweg bei Verbindungsabbruch und b) das Vorgehen bei ggf. aufkommender Eigen- und/oder Fremdgefährdung zu vereinbaren. Nach dem Patientenrechtegesetz (§ 630e BGB) muss die mündliche Aufklärung des Patienten erfolgen und die Einwilligung zur Videobehandlung eingeholt werden.

Psychotherapeuten können ambulante Leistungen in Deutschland nur abrechnen, wenn sie der kassenärztlichen Vereinigung zuvor angezeigt haben, einen nach Anlage 31b des Bundesmantelvertrages-Ärzte zertifizierten Videodienstanbieter einzusetzen, wobei derzeit regionale Ausnahmeregelungen gelten (https://www.kbv.de/html/videosprechstunde.php). Diese Anbieter gewährleisten die Ende-zu-Ende-Verschlüsselung und realisieren die Konsultationen über eine browserbasierte Software, die auf Desktop-PC, Laptops, Tablets und Smartphones genutzt werden kann. Erfahrungsgemäß empfinden Patienten den Bildausschnitt von Smartphones jedoch für Videokonsultationen als zu klein (Bleyel et al. 2020). Folgende technische Voraussetzungen sollten erfüllt sein:

- Webcam (idealerweise mit einer Auflösung >720 p) und Mikrofon,
- stabile Internetverbindung mit Download-Bandbreite >4 Mbit/s (mindestens 384 Kbit/s) und Upload-Bandbreite >1 Mbit/s (prüfbar beispielsweise über https://breitbandmessung.de), über Local Area Network (LAN), Wireless Local Area Network (WLAN) und mobilen WLAN-Hotspot (Wi-Fi Hotspot) im Mobilfunknetz.

Zu Beginn der Videokonsultation muss ggf. dem Browser die Berechtigung zur Nutzung von Kamera und Mikrofon erteilt werden. Während der Videokonsultation sollte keine Werbung erscheinen. Bei Nutzung eines Desktop-PC sollte die Webcam oberhalb des Bildschirms und so nah wie möglich am Bild des Patienten auf dem Bildschirm platziert sein, um den Blickwinkel zu reduzieren und ein Gefühl von Augenkontakt mit dem Patienten herzustellen. Zur Prüfung von Auftreten und Haltung sollte der Psychotherapeut routinemäßig die Bild-in-Bild-Funktion nutzen. Um die Bandbreite nicht unnötig zu beanspruchen, wird empfohlen, auf das Tragen von greller (etwa weißer oder grüner) Kleidung oder solcher mit optisch irritierenden Mustern (beispielsweise Krawattenmuster oder Schals) zu verzichten. Zudem sollte ggf. getragener Schmuck auf mögliche Blendeffekte hin überprüft werden.

Mit dem Patienten sollte geklärt werden, ob die Tonqualität für ihn stimmig ist. Etwaige Nebengeräusche sollten bestmöglich reduziert werden. Für die optimale akustische Qualität wird empfohlen, ein Headset zu nutzen und die Lautstärke nicht zu hoch einzustellen. *Eine mittlere Lautstärkeneinstellung verringert den Echoeffekt* – eines der häufigsten initialen Probleme bei Videokonsultationen. Sollte dennoch ein Echoeffekt auftreten, hilft es meist, die Lautstärke zu mindern (zunächst patienten-, dann auch therapeutenseitig). Häufig besteht bei Videokonsultationen die Tendenz, besonders laut zu sprechen. Das kann zu Irritationen führen und ist überwiegend nicht nötig. Sollten die Beteiligten „übereinander sprechen“, sollte der Psychotherapeut nonverbal signalisieren, dass er zuhört (beispielsweise durch eine Handgeste oder Zeigen auf sein Ohr). Bei unvorhergesehenen Störungen aufseiten des Psychotherapeuten sollte dieser sein Mikrofon stummschalten. Häufige technische Schwierigkeiten sind die nichtgelungene Einwahl zum Videodienst aufseiten des Patienten, das Verpixeln und/oder Einfrieren des Bildes sowie Verzögerungen und Verbindungsabbrüche in der Bild- und/oder Audioübertragung (Greenhalgh et al. 2018).

Der Raum, der vom Psychotherapeuten für die Videokonsultationen genutzt wird, sollte in allererster Linie geschützt sein, sodass diese störungsfrei stattfinden können. Bei Räumen, die bisher noch nicht für Patientenkontakte genutzt wurden, ist besonders sorgfältig auf die entsprechende Eignung (beispielsweise nicht zu dünne Wände) zu achten. Es sollte sichergestellt sein, dass während der Konsultation keine weitere Person diesen Raum betritt und dass von außen nicht mitgehört werden kann. Um Ablenkungen und störende Geräuschquellen zu vermeiden, müssen Türen und Fenster geschlossen sein. Externe Lärmquellen wie etwa aus Nebenzimmern oder von Baustellen sollten, wenn möglich, minimiert werden. Auch der vom Patienten verwendete Raum sollte geschützt sein. Mithören durch Dritte muss daher vom Patienten verhindert werden. Der Psychotherapeut sollte den Patienten stets darauf hinweisen, dass er dies sicherstellt.

Im Blickfeld der Webcam sollte keine (politisch/religiös) polarisierende Dekoration sichtbar sein. Auf einen möglichst neutralen Hintergrund ist zu achten. Der Raum sollte gleichmäßig gut ausgeleuchtet sein. Empfohlen werden Lichtquellen an der Raumdecke, um Schattenbildungen zu vermeiden. Durch einseitige Beleuchtung können große Schatten auf einer Seite des Gesichts liegen. Direkt gegenüber der Kamera sollte sich kein Fenster befinden, da das Gesicht durch das Tageslicht auf dem Bildschirm sehr dunkel wird und nicht mehr zu erkennen ist. Der Psychotherapeut sollte sicherstellen, dass er selbst und der Patient die richtige Position einnehmen bzw. in dieser verbleiben. Die angemessene Distanz zum Bildschirm beträgt ca. 50 cm bei Tablets und 100 cm bei Desktop-PC. Während der Konsultation sollte der Eindruck von Augenkontakt entstehen. Eine leicht nach vorn gelehnte Sitzhaltung seitens des Psychotherapeuten vermittelt einen höheren Grad an Empathie und Vertrautheit. Dabei sollte der Blick nach vorn frontal zu der Webcam gerichtet sein. Die Augen sollten auf Position der Trennlinie zwischen dem oberen und den beiden unteren Dritteln des Bildschirms gerichtet sein (evtl. kann eine kleine Markierung angebracht werden). Dadurch wird ein möglichst guter Eindruck von Augenkontakt gewährleistet. Um Gesichtsausdrücke besser wahrnehmen zu können, sollte die Patientin/der Patient – soweit angemessen – ihre/seine Kopfbedeckung abnehmen. Die Anmeldung des Psychotherapeuten beim Videodienstanbieter erfolgt mit Angabe der E‑Mail-Adresse und des Passworts unmittelbar vor Beginn einer Videokonsultation über die entsprechende Website. Der Patient wählt sich beispielsweise über eine bei Erstkontakt vom Psychotherapeuten generierte und telefonisch übermittelte Transaktionsnummer ein.

Sobald die Videokonsultation begonnen hat, sollte jegliche verbale und nonverbale Information bereits unter diagnostischen/therapeutischen Gesichtspunkten betrachtet werden. Patienten sollten die Bild-in-Bild-Option deaktivieren und darauf hingewiesen werden. Zu Beginn jeder Videokonsultation sollte der Psychotherapeut den genauen Ort erfragen, an dem sich der Patient befindet, und mit ihm klären, ob die Angaben im Notfallplan weiterhin gültig sind, und wer an der Videokonsultation teilnimmt. Jeder Teilnehmer sollte stets auf dem Bildschirm zu sehen sein. Patient und Psychotherapeut sollten sich ggf. gegenseitig versichern, dass keine anderen Personen im Raum sind bzw. den Raum während der Konsultation betreten (Morland et al. 2015). Gegebenenfalls kann der Psychotherapeut kurz die Kamera durch seinen Raum schwenken, sodass sich der Patient vergewissern kann, dass niemand anderes zugegen ist. In umgekehrter Weise sollte sich der Psychotherapeut beim Patienten vergewissern können.

Werden die angeführten Aspekte berücksichtigt, sind gute Voraussetzungen für die Durchführung von psychotherapeutischen Videokonsultationen gegeben. Sollten Schwierigkeiten auftreten, sollte der Psychotherapeut einen einmaligen Wiederverbindungsversuch unternehmen und, wenn dieser erfolglos verläuft, *rasch *auf das Telefon umsteigen (Shore et al. 2018).

## Erfahrungen in einer deutschen Machbarkeitsstudie

Im vom Bundesministerium für Bildung und Forschung (BMBF) durchgeführten PROVIDE-Projekt (https://www.provide-project.de) werden Videokonsultationen durch Psychotherapeuten bei in der Hausarztpraxis vorstelligen Patienten mit Depressionen und Angststörungen erprobt (Haun et al. 2019; Tönnies et al. 2019). Im Rahmen der Prozessevaluation der Machbarkeitsstudie wurden neben der quantitativen Erhebung der Praktikabilität auch leitfadengestützte Interviews mit 20 der 23 Patienten der Interventionsgruppe (mittlere Dauer: 24 min) und den 3 Studientherapeuten geführt (43 min). Im Folgenden wird ein Einblick in die Ergebnisse gegeben.

Insgesamt bewerteten die Studientherapeuten die Videokonsultationen als praxistauglich und gut durchführbar, jedoch ausnahmslos auch als „anstrengender“ (PT1:51). Trotz anfänglicher Bedenken, insbesondere hinsichtlich der Etablierung einer tragfähigen therapeutischen Beziehung, waren alle Therapeuten nach einem Gewöhnungsprozess von der Machbarkeit der Videokonsultationen überzeugt. Für eine Studientherapeutin war „dieser Computer kein Hinderungsgrund“ (PT3:107), eine andere meinte, „Übertragungs- und Gegenübertragungsphänomene [hätten] auch einfach sich schnell im Video gezeigt“ (PT2:92–93). Mit punktuellen technischen Störungen hatten alle Studientherapeuten zu kämpfen. Die Antizipation von Unterbrechungen oder Verzögerungen der Ton- und/oder Bildübertragung ging für 2 der 3 Studientherapeuten mit einer spezifischen Unsicherheit im therapeutischen Gespräch einher. Die fehlende Leiblichkeit und persönliche Interaktion wurden von allen Therapeuten als deutlichster Nachteil benannt: „Wenn man den Patienten sieht, dem z. B. nicht die Hand zu geben. Das finde ich immer eine komische Situation … ich finde jetzt dieses Händeschütteln und dieser körperliche Kontakt, der dann ja trotzdem immer in jeder Stunde mit den Patienten da ist, der fehlt“ (PT2:68–75).

Die Patienten berichteten von einem aus ihrer Sicht weitestgehend reibungslosen Ablauf der einzelnen Videokonsultationen. Acht Patienten beschrieben Skepsis bzw. zurückhaltende Erwartungen vor Beginn der ersten Videokonsultation, was sich jedoch meist schon nach der ersten Sitzung relativierte. Insgesamt gaben fast alle Patienten an, von den Videokonsultationen profitiert zu haben (19 von 20 Patienten). Die wesentlichen Nachteile bezogen sich auf Unterbrechungen der Bild- und/oder Tonübertragung (17/20). Eine Patientin empfand diese als „extrem störend in so einer Situation, also eine gute Verbindung ist, glaube ich, das Muss an so einem Termin“ (I20:2). Die fehlende persönliche Interaktion empfanden nur 5 Patienten als Nachteil. Alle Patienten nahmen die therapeutische Beziehung als positiv und hilfreich wahr (20/20): „Das Gespräch mit dem Therapeuten an sich … das war dann schon, sag ich mal, ein bisschen lösend oder auch befreiend“ (I3:33–34). Der Modus Video scheint für die Patienten eine untergeordnete Rolle zu spielen; die räumliche Distanz habe sich „zunehmend verwischt; dass es da eine räumliche Distanz gibt, eine echte Realität, das ist dann nicht mehr so bedeutsam gewesen“ (I1:461–464). Einige Patienten empfanden es als „einfacher“ (I8:42) und „angenehmer“ (I14:74), sich im Gespräch über Video zu öffnen, im Vergleich zur persönlichen Therapie (4/20). Ferner hob die Hälfte der Patienten eingesparte Wegzeiten als Vorteil hervor (10/20).

In weitgehender Übereinstimmung mit Simpson und Reid (2014) machen die vorgestellten Ergebnisse deutlich, dass Patienten in videobasierten Psychotherapien ein tragfähiges Arbeitsbündnis und insbesondere ein Gefühl von Präsenz erleben. Auch in der vorgestellten Studie erlebten die Psychotherapeuten die Allianz zu Beginn der Therapie tendenziell zunächst als weniger belastbar. Die wesentlichen Verbesserungsvorschläge von Patienten und Psychotherapeuten bezogen sich auf die Stabilität der Internetverbindung. War diese gegeben, konnte in vielen Fällen ein tragfähiges Arbeitsbündnis etabliert werden.

## Anwendung am Beispiel der Routineversorgung im Krankenhaus

Auch im Krankenhaus ist durch die COVID-19-Pandemie die Bedeutung von Videokonsultationen sprunghaft angestiegen. Insbesondere bei somatisch schwer kranken Risikopatienten ist eine Abwägung von psychosozialem Bedarf und Infektionsschutz geboten. Exemplarisch wird im Folgenden über die Anpassung in der psychoonkologischen Ambulanz am Nationalen Centrum für Tumorerkrankungen (NCT) Heidelberg berichtet. Um einerseits den z. T. gestiegenen Bedarf nach Gesprächen zu gewährleisten und andererseits die besonders gefährdeten Patienten keinen zusätzlichen Risiken durch eine Infektion mit dem SARS-CoV-2-Virus auszusetzen, werden derzeit Patienten auch videobasierte Beratungen angeboten. Von 180 Gesprächen im Zeitraum vom 17.03.2020 bis zum 30.04.2020 fanden 27 als Videogespräch statt (15 %), während 130 Beratungen per Telefon durchgeführt wurden und 23 im persönlichen Kontakt erfolgten. Offensichtlich müssen sich Therapeuten und Patienten zunächst an das neue Setting gewöhnen. Der Videoanteil stieg während der 6 Wochen kontinuierlich an. Alle Patienten, die einmal mit Videokonsultationen begonnen hatten, führten auch Folgekontakte in dieser Weise fort. Informelle Rückmeldungen von Therapeuten und Patienten decken sich mit den Interviewbefunden der PROVIDE-Studie. Allerdings zeigen sich auch Grenzen: So erleben die Therapeuten z. B. Paarbegleitungen per Video bei starken Differenzen deutlich schwieriger. Aktuell werden Versuche durchgeführt, eine geplante Gruppentherapie auf ein Videoformat umzustellen, wofür eine erhebliche Motivationsarbeit bei den Patienten nötig ist. In den meisten Fällen überwiegen jedoch die positive Bilanz und die Dankbarkeit der sozial stark isolierten Patienten mit Tumorerkrankungen, denen über das Medium Video der persönliche Kontakt ohne Ansteckungsgefahr möglich ist. Mit zunehmenden Lockerungen wird jedoch auch der Wunsch nach persönlichen Kontakten wieder größer.

Einschränkend soll angefügt sein, dass die geschilderten Erfahrungen exemplarischen Charakter haben und in systematischen Beobachtungsstudien (beispielsweise idealerweise repräsentativen Befragungen von Patienten und Professionellen) überprüft werden sollten.

## Fazit für die Praxis

- Videokonsultationen wird über die „Coronavirus-disease-2019“(COVID-19)-Pandemie hinaus künftig in der Routineversorgung mehr Bedeutung zukommen, um v. a. mobilitätseingeschränkte Patienten (beispielsweise durch körperliche Komorbidität) und Patienten in ländlichen oder entlegenen Gebieten mit geringer psychotherapeutischer Dichte zu erreichen.
- Hinsichtlich der Wirksamkeit zeigen sich bisher keine Unterschiede zwischen videobasierten Interventionen und klassischer Angesicht-zu-Angesicht-Therapie, wobei Nichtunterlegenheitsstudien größtenteils fehlen und die Generalisierbarkeit stärker untersucht werden sollte.
- Zwar sind bei der Implementierung von Videokonsultationen wenige Setting-spezifische Aspekte zu beachten. Stabile Konnektivität vorausgesetzt, kann jedoch ein tragfähiges therapeutisches Arbeitsbündnis aufgebaut werden.
- Erste Ergebnisse aus einer deutschen Machbarkeitsstudie und klinischer Anwendung während der COVID-19-Pandemie weisen auf die gute Praktikabilität und Akzeptanz bei Psychotherapeuten und Patienten hin.
